# IL-4 Attenuates Pulmonary Epithelial Cell-Mediated Suppression of T Cell Priming

**DOI:** 10.1371/journal.pone.0045916

**Published:** 2012-09-20

**Authors:** Melanie Albrecht, Markus Arnhold, Sandra Lingner, Subhashree Mahapatra, Dunja Bruder, Gesine Hansen, Anna-Maria Dittrich

**Affiliations:** 1 Pediatric Pneumology, Allergology and Neonatology, Medical School Hannover, Hannover, Germany; 2 Immune Regulation Group, Helmholtz Centre for Infection Research, Braunschweig, Germany; 3 Research Group Infection Immunology, Department of Medical Microbiology, Otto-von-Guericke-University Magdeburg, Magdeburg, Germany; Leiden University Medical Center, The Netherlands

## Abstract

We have previously shown that Th2-polarized airway inflammation facilitates sensitization towards new, protein antigens. In this context, we could demonstrate that IL-4 needs to act on cells of the hematopoetic and the structural compartment in order to facilitate sensitization towards new antigens. We thus aimed to elucidate possible mechanisms of action of IL-4 on structural cells choosing to analyze pulmonary epithelial cells as an important part of the lung's structural system. We used a co-culture system of DC- or APC-dependent *in vitro* priming of T cells, co-cultivated on a layer of cells of a murine pulmonary epithelial cell line (LA-4) pretreated with or without IL-4. Effects on T cell priming were analyzed via CFSE-dilution and flow cytometric assessment of activation status. Pulmonary epithelial cells suppressed T cell proliferation *in vitro* but this effect was attenuated by pre-treatment of the epithelial cells with IL-4. Transwell experiments suggest that epithelial-mediated suppression of T cell activation is mostly cell-contact dependent and leads to attenuation in an early naive T cell phenotype. Secretion of soluble factors like TARC, TSLP, GM-CSF and CCL20 by epithelial cells did not change after IL-4 treatment. However, analysis of co-stimulatory expression on pulmonary epithelial cells revealed that pre-treatment of epithelial cells with IL-4 changed expression GITR-L, suggesting a possible mechanism for the effects observed. Our studies provide new insight into the role of IL-4 during the early phases of pulmonary sensitization: The inhibitory activity of pulmonary epithelial cells in homeostasis is reversed in the presence of IL-4, which is secreted in the context of Th2-dominated allergic airway inflammation. This mechanism might serve to explain facilitated sensitization in the clinical context of polysensitization where due to a pre-existing sensitization increased levels of IL-4 in the airways might facilitate T cell priming towards new antigens.

## Introduction

For a long time, pulmonary epithelial cells were thought to exert a fundamental role as a barrier towards deleterious substances only. However, recent advances have highlighted crucial effects of epithelial cells on the modulation of an immune response in general, and allergic airway disease in particular.

In this context epithelial cells have been shown to exert direct and indirect effects on T cell function during allergic airway disease. Human tracheal, bronchial and alveolar epithelial cell have been shown to express various members of the B7 family whose expression is modulated by viral infection and cytokines [1]. Moreover, the secretion of IL-4 and-13 during a Th2-polarized immune response serves as an amplification signal via epithelial cells for the Th2 response as these cytokines induce the secretion of various chemokines, such as RANTES, MCP-1[2], thymus and activation-regulated chemokine (TARC) [3] and eotaxin [4] from epithelial cells, which leads to further recruitment of Th2 cells. Furthermore, by secretion of interleukin-1F9 (IL-1F9), and interleukin-33 (IL-33), epithelial cells can directly amplify Th2 polarization via the ST2 receptor [5]. By means of indirect action, epithelial cells have additional effects on the course of a T cell response: epithelial cells increase dendritic cell recruitment and survival by secretion of CCL20 and GM-CSF [6], [7] which in turn can influence T cell activation and differentiation. Additionally, epithelial cells affect the activation of APC: binding of double-stranded RNA, or Th2 cytokines lead to the production of thymic stromal lymphopoietin (TSLP) by epithelial cells [8]. TSLP, in turn, modulates the expression of CD40, CD80 and OX40L on DCs thereby promoting Th2 polarization [9], [10].

It has been recognized for some time that in homeostasis, in spite of MHCII expression, airway epithelial cells rather than promoting T cell activation, induce hyporesponsiveness of T cells [11]. However, only more recent studies demonstrated that epithelial cells from colon but also from the airways not only fail to activate T cells but suppress APC-induced T cell activation [12], [13]. Yet, the mechanisms underlying this effect remain controversial with unequivocal results regarding the role of soluble and cell-surface bound mediators and the role of regulatory T cells.

This selection of recent findings on the interaction of airway epithelial cells with T cells shows that in addition to the barrier function the epithelium may exert important immunomodulatory functions that affect the adaptive immune response in the airways. It also demonstrates that several open questions remain with regards to the interactions between epithelial cells and T cells.

Immunomodulatory properties of the airway epithelium might also play a role in the mechanisms underlying the clinical phenomenon of polysensitization in asthma, which refers to patients who after being sensitized to one antigen are at higher risk to acquire new sensitizations to harmless antigens and thus become sensitized to various, unrelated antigens [14], [15].

We have recently shown that the adjuvant effects that IL-4 exerts on allergic sensitization via the airways depend on the ability of structural cells to respond to IL-4Rα signalling [16]. To analyze the effect of IL-4 on epithelial cells, which constitute an important component of the structural cell compartment in the lung, we used an in in vitro co-culture system of APC, TC and a murine pulmonary epithelial cell line. With this co-culture system, we could recapitulate older studies which demonstrate that airway epithelial cells suppress APC-dependent T cell activation [13]. Interestingly, in our hands, this effect is almost exquisitely cell-contact dependent but does not lead to the generation of regulatory T cells. Pre-treatment of the airway epithelial cells, which were shown to express the IL-4Rα, dramatically reduces their suppressive effect. We were thus able to identify epithelial cells as a target of IL-4 mediated allergic sensitization.

## Materials and Methods

### Mice

BALB/cJ (WT) and C.129S2-*Stat6^tm1Gru^*/J (STAT6 KO) mice were purchased from The Jackson Laboratory. TCR-transgenic CD4^+^ T cell donors were DO11.10 mice backcrossed onto a BALB/c αß^−/−^ background and were bred in at the Institute for Laboratory Animal Science, Hannover Medical School (MHH), Germany. Six-to 10-wk-old female mice were used in all experiments. All experimental methods described in this manuscript were in accordance with the German Animal Welfare Legislation and performed as approved by the Lower Saxony State Office for Consumer Protection and Food Safety (LAVES; application no. 33.9-42502-04-07/1369).

### BM transfers

Bone marrow cells were isolated from both femur and tibia of male mice. Cells were treated with red blood cell lysis medium (1.6 g NH_4_Cl, 0.2 g KHCO_3_, and 0.03 g EDTA) for 5 minutes, washed and injected at 5–8×10^6^ BM cells/mouse into congenic, irradiated (2×600 rad, 4 hours apart) female recipient mice. Eight weeks were allowed for reconstitution.

### Airway sensitization and challenge

For sensitization via the airways mice were exposed to 5 µg of ovalbumin (OVA) (grade V; Sigma-Aldrich) + 1 µg IL-4 (BD Biosciences) intranasally on days 0, 1 and 2. Challenge was performed with 25 µg of OVA on days 14, 15, 18 and 19. For analysis of bronchoalveolar lavage (BAL), OVA-specific IgE and mediastinal lymph node cytokine production, mice were sacrificed on day 21. Control mice for all experiments were treated with OVA alone, challenge was performed as outlined above.

### Analysis of BAL

BAL inflammatory cells were obtained by cannulation of the trachea and lavage of the airway lumen with 1ml PBS. Cytospin slides were stained with Diff-Quick (Dade Behring) and 200 cells/sample were differentiated microscopically.

### Determination of serum antibody concentration

For measurement of OVA-specific IgE antibodies, plates were coated with 100 µl of 2 µg/ml anti-mouse IgE (R35–72, BD Biosciences) at RT for 4 hours. Assay Diluent Buffer (BD Biosciences) was used to block plates. Sera were incubated overnight at 4°C followed by digoxigenin-OVA (made in our own laboratory) and digoxigenin-conjugated POD (F. Hoffmann-La Roche) at RT, followed by tetramethylbenzidine substrate (DAKO). IgE standard was generated by hyper-immunization of mice with OVA/Alum i.p. for six consecutive times, highest standard dilution was set at 500 U/ml with detection limit being 31.25 U/ml.

### Lymph node (LN) preparation

Mediastinal LNs were harvested and pooled from each group at time of sacrifice. Single cell suspensions were obtained by mechanical disruption. Cells were either stimulated in vitro with 200 µg/ml OVA and syngeneic T cell-depleted splenocytes.

### Isolation of CD4+ T cells

CD4^+^ T cells were isolated from the spleens of transgenic mice by negative selection using antibodies to MHC class II I–A^d^ (212.A1), CD8 (TIB 210), B220 (TIB 164), and FcR (24G2) (all prepared and purified in our lab) followed by anti-Ig-coated magnetic beads (Polysciences). A fraction of CD4^+^ T cells were subjected to FACS analysis for purity control with purities typically ranging from 85–90% KJ1-26/CD4+ T cells.

### BMDC preparation

For generation of bone marrow derived dendritic cells, bone marrow cells were isolated by perfusion of femur and tibia. Cells were cultured in RPMI 1640 (Invitrogen Corp.) in the presence of 1% culture supernatant from a cell line transfected with the murine GM-CSF gene. After 6 days, cells were harvested and a fraction of DCs were subjected to FACS analysis for purity control with MHCII/CD11c+ cells constituting between 60–70% of cells analyzed.

### APC preparation

Syngeneic T cell-depleted splenocytes were prepared by Ab-mediated rabbit complement lysis using Abs to CD4 (GK1.5) and CD8 (TIB 105) (all prepared and purified in our lab) in conjunction with mitomycin C (Sigma-Aldrich) treatment (50 µg/ml for 30min at 37°C) in order to prevent proliferation of these cells. A fraction of splenocytes were subjected to FACS analysis for purity control with MHCII-expressing cells constituting 80–90% of cells and 5–8% of contaminating CD4+ and CD8+ cells.

### LA4 Culture

The LA4 cell line, derived from a murine lung adenoma, shows characteristics of type II pneumocytes [17] and was obtained from the European Collection of Cell Cultures (ECACC). LA4 cells were cultured in F12K nutrient mixture (Lonza) with 10% FCS, 2 mM L-glutamine (Sigma-Aldrich), 100 U/mL penicillin and 0.1 mg/mL streptomycin (Sigma-Aldrich). They were passaged two to three times/week splitting 1∶2 to 1∶3 depending of confluency. To this end, cells were trypsinized (0.03–0.04 ml/cm^2^ trypsin/EDTA) for three minutes after which the reaction was stopped by an excess of culture medium, cells were harvested, counted and split as outlined above.

### Co-cultures

- Transgenic T cells and DC were co-cultured in a ratio of 5∶1, DCs being pre-pulsed with 4,5 mg/ml whole OVA protein (grade V, Sigma-Aldrich) 24 h before co-culture. Before addition to T cells for co-culture, DCs were extensively washed to remove excess OVA protein not taken up and processed by the DCs.
- Transgenic T cells and APC were co-cultured at a ratio of 2∶1 with the addition of OVA-peptide, pOVA (5 µg/ml, Biosyntan, OVA_323–339_, aa sequence: ISQAVHAAHAEINEAGR) immediately before co-culture.

Cultures were performed on a confluent layer of LA4 cells in 12- or 24-well flat-bottom plates. T cell proliferation was determined by CFSE-dilution on day 3–7 as indicated in figure legends. 30,000–40,000 CD4+ cells in each sample were analyzed for CFSE intensity by flow cytometry with a fixed number for all samples in a given experiment. All cells having a lower CFSE intensity than non-proliferated control cells were designated as “CFSElow” cells.

### IL-4 treatment

Recombinant murine IL-4 (BD Biosciences) was added to LA4 cell cultures 24 h before co-culture with T cell and DC/APC was initiated at the indicated doses. Prior to addition of T cells and DC/APC, LA4 cells were washed 5 times with complete medium in order to ensure complete removal of IL-4 in culture supernatants.

### Trypan blue staining

LA4 cells were treated with different amounts of IL-4 (2–200 ng/ml) for 2–24 h. Harvested cells were stained with trypanblue solution (0.5% in PBS; Carl Roth) and analyzed microscopically for percentage of viable cells (trypan blue exclusion).

### CFSE labelling

OVA-specific T cells were labelled with 5 µM of CFSE (eBioscience) in PBS/0.1% BSA for 10 minutes at 37°C. Labelling was stopped by adding ice-cold cell culture medium.

### Flow cytometry analysis

All staining procedures were performed on ice. Cells were incubated with anti-FcR (24G2) antibody in combination with mouse Ig for 20 minutes on ice, then stained with antibodies against CD11c, CD11b, MHC class II, CD4, CD8, CD44, CD62L, CD25, CD69 and appropriate isotype controls (BD Biosciences) for 30 minutes on ice. The presence of OVA-specific T cells was determined by staining with an anti-clonotypic, self-grown mAb (KJ1-26) antibody. Intracellular FoxP3 staining was determined after staining with a FoxP3 staining kit (eBioscience) according to the manufacturer's recommendations. CFSE dilution was determined in the FITC channel. Cells were analyzed on a LSRII (Becton Dickinson) flow cytometer in association with FlowJo (Treestar) software.

### Immunohistochemistry Staining

LA4 cells or splenocytes were cytospun (Shandon cytocentrifuge, ThermoShandon, 500 g, 5′), blocked with 1.5% skimmed milk and consecutively incubated with a biotinylated rat-anti-mouse CD124 (IL-4Rα) (clone: M1; BD Pharmingen) antibody or the appropriate isotype control (PE-rat IgG2a, BD-Pharmingen), developed via addition of Streptavidin-FITC (eBioscience) and visualized on a fluorescent microscope at the appropriate wavelength.

### Cyto- and chemokine measurements

Cytokines from LN cultures of bone marrow chimeras were determined by Multiplex technology (Millipore) according to the manufacturer's recommendations. TGF-β, TARC, TSLP, GM-CSF, CCL20, IL-2 and IFN-gamma in supernatants of co-cultures or IL-4 treated LA4-cells were determined via DuoSet ELISAs (R&D Systems) according to the manufacturer's instructions.

### RNA Isolation, cDNA Sythesis and PCR

Total RNA was isolated from LA4 cells using an RNA isolation kit from Qiagen as described by the manufacturer. Synthesis of cDNA was accomplished by using the first strand cDNA synthesis kit from Fermentas according to the manufacturer's instructions. IL-4Rα was amplified by Taq-Polymerase (Fermentas) with dNTPs (Fermentas), isolated cDNA and IL-4Rα (CD124)-specific primers (forward primer: TGT GCC ACA TGG AAA TGA AT, reverse primer: CAT TGG TGT GGA GTG TGA GG) with a PCR-programm adapted to these primers on the Mastercycler epGradient (Eppendorf), resulting in a fragment of 150 bp which were run on a 2% agarose gel.

### Determination of statistical significance

Statistical significance was determined using the Mann-Whitney *U* test if not stated otherwise in the figure legends. A *p*<0.05 was considered to be significant. Unless indicated otherwise, five mice were used for each condition studied in an individual experiment. The number of repeat experiments is disclosed in the individual figure legends.

## Results

### IL-4 facilitates airway sensitization by acting on hematopoetic and structural cells

**Figure 1:**
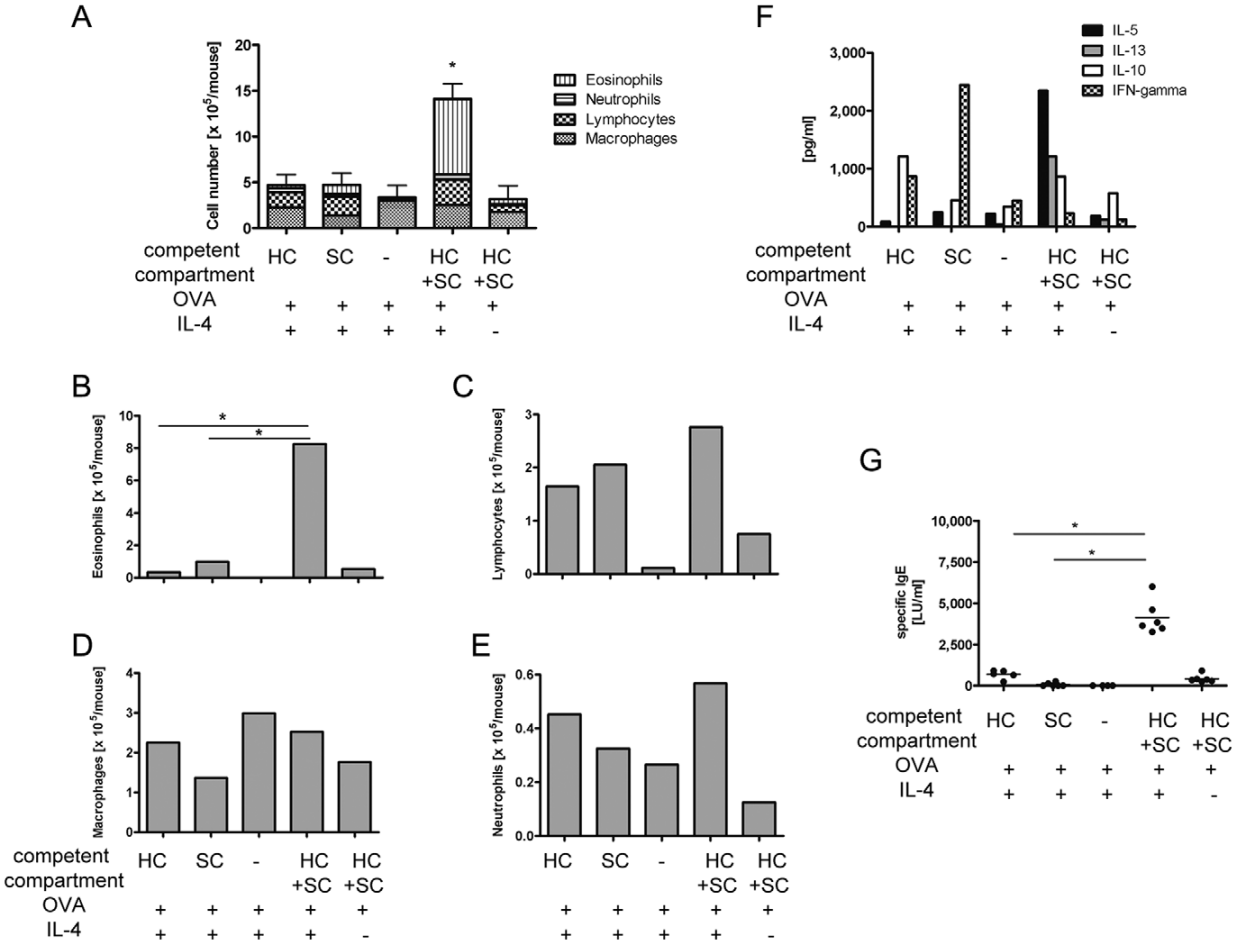
IL-4 facilitates airway sensitization by acting on hematopoetic and structural cells. Chimeric mice, expressing STAT6 in either SC, HC, neither or both were sensitized intranasally with 5 µg OVA in combination with 1 µg IL-4 for three consecutive days and challenged with 25 µg OVA for four days. Animals sensitized with OVA only served as controls. Analyses were performed two days after last challenge. A: BAL cell differentiation of STAT6 chimeric mice. B–E: BAL cell distribution of STAT6 chimeric mice, B  =  eosinophils, C =  lymphocytes, D =  macrophages, E =  neutrophils. F: OVA-specific cytokine expression in lung draining lymph nodes from STAT6 chimeric mice on day 3 of culture. G: OVA-specific IgE in serum of STAT6 chimeric mice. SC  =  structural compartment. HC  =  hematopoetic compartment. LU  =  laboratory units. *p<0.05 by Mann-Whitney-U-test compared to all other variables. Representative experiments of n = 2–8 experiments.

In order to dissect the contribution of structural and hematopoetic cells towards IL-4 dependent airway sensitization, we created bone marrow chimeras expressing the transcriptions factor STAT6 in either the structural compartment or the hematopoetic compartment only, neither compartment (corresponding to a knock-out mouse) or on both compartments (Figure 1). STAT6 is the critical transcription factor for IL-4-mediated signalling and thus its expression constitutes a prerequisite for IL-4 dependent signalling. After reconstitution these mice were subjected to intranasal sensitization with a subimmunogenic dose of ovalbumin (OVA) (titrated in previous experiments) and IL-4, a protocol where priming depends on the addition of the IL-4 [16]. Similar to our previous results with bone marrow chimeras for the α-subunit of the IL-4 receptor [16], IL-4-dependent pulmonary priming depends on the expression of STAT6 in both the structural and the hematopoetic compartment (HC+SC in figure legends of Figure 1) in order to allow development of eosinophilic airway inflammation, Th2 cytokine secretion and antigen-specific IgE secretion, hallmarks of the full phenotype of Th2-polarized airway inflammation. Lack of STAT6 in the hematopoetic or the structural compartment reduced airway inflammation in both cases (1^st^ and 2^nd^ bar, Figure 1A) to levels similar to those observed in mice which did not express the transcription factor in either compartment (3^rd^ bar, Figure 1A) while expression in both compartments (4^th^ bar, Figure 1A) allowed significant Th2-polarized airway inflammation. Senzitization with OVA alone (without IL-4) also did not induce significant airway inflammation (5^th^ bar, Figure 1A), confirming that the doses of OVA used were indeed subimmunogenic. The reduction of airway inflammation was due to a significant decrease in eosinophils (Figure 1B). Lymphocytes (Figure 1C), macrophages (Figure 1D) and neutrophils (Figure 1E) were also reduced in the bronchoalveolar fluid (BALF) of mice which expressed STAT6 on the hematopoetic cells or the structural cells only, however, these reductions failed to attain statistical significance.

We observed a similar picture when analyzing antigen-specific cytokine secretion by draining lymph node (LN) cells from these mice. Expression of STAT6 on hematopoetic or structural cells only (1^st^ and 2^nd^ set of bars in Figure 1F) led to a reduction in the Th2 cytokines IL-5 and-13 and also IL-10 secretion compared to secretion by the LN cells of mice who expressed the transcription factor on the hematopoetic cells and the structural cells (4^th^ set of bars in Figure 1G). Interestingly, if STAT6 was expressed on structural cells only (2^nd^ set of bars in Figure 1G) IFN-gamma was super-induced to levels not observed in any other group.

Finally, deletion of STAT6 on either the structural compartment (1^st^ bar, Figure 1G) or the hematopoetic compartment (2^nd^ bar, Figure 1G) also significantly reduced secretion of antigen-specific serum IgE compared to secretion from animals expressing the receptor in both compartments (4^th^ bar, Figure 1G).

In summary, these results support our previous findings that IL-4 dependent priming depends on IL-4 signalling in not only hematopoetic but also structural cells [16], a finding which we consecutively analyzed in more detail by the experiments described below.

### Airway epithelial cells suppress antigen-specific T cell proliferation

Our observation that IL-4 signalling needs to take place not only in hematopoetic cells but also in structural cells in order to allow significant sensitization and airway inflammation led us to hypothesize that IL-4 can modulate pulmonary epithelial cell function. To investigate this hypothesis we decided to use a co-culture system of DC-induced antigen-specific T cell activation. Prior to exploring the influence of IL-4 treatment on pulmonary epithelial cells in this co-culture system, we carefully characterized the effects of the pulmonary epithelial cell line LA4 on T cell activation per se in order to determine which parameters our investigations of IL-4-mediated effects should include.

**Figure 2:**
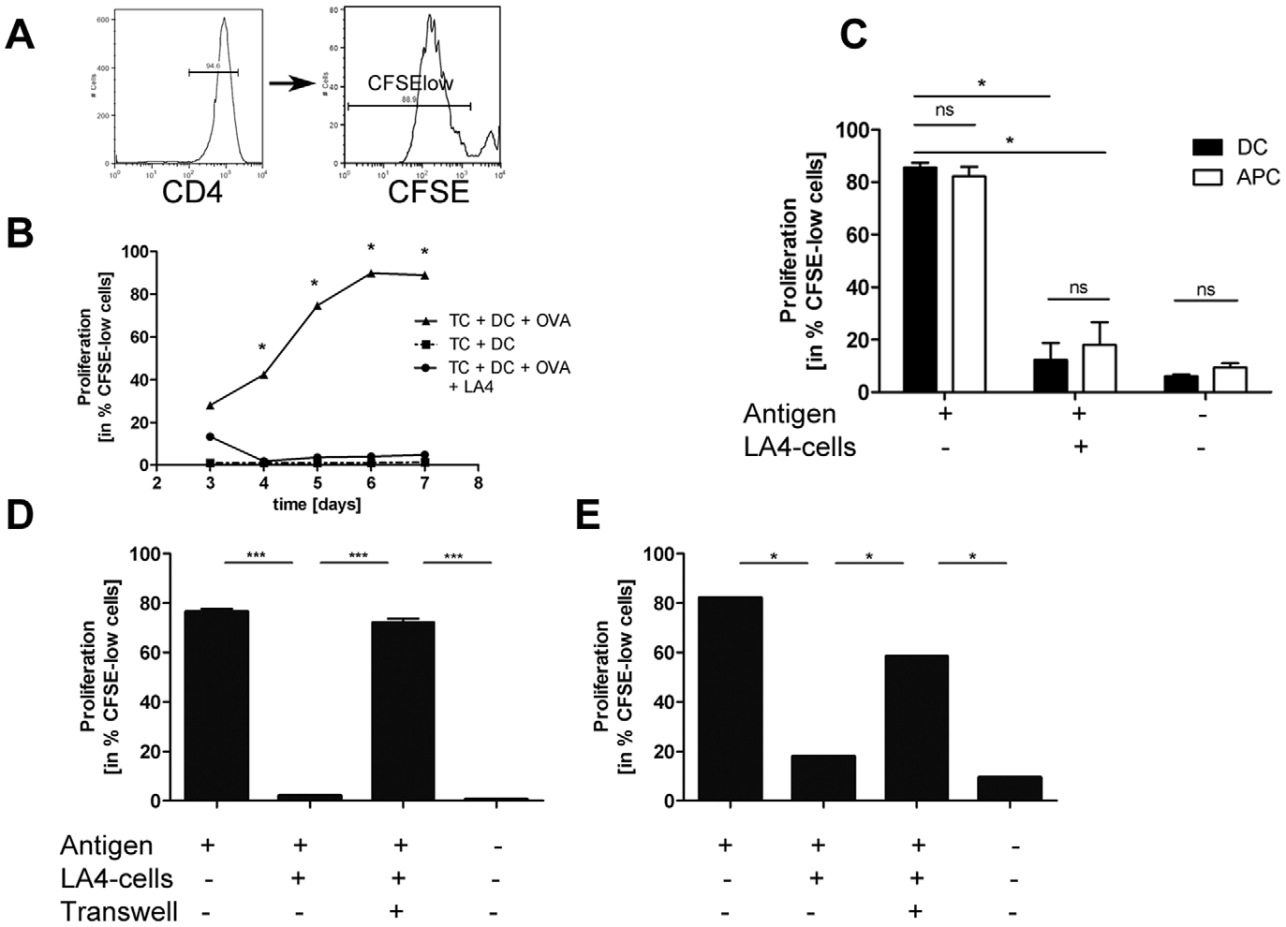
Airway epithelial cells suppress antigen-specific T cell proliferation. A: Gating strategy for assessment of T cell proliferation by CFSE dilution. All CD4+ cells having a lower CFSE intensity than non-proliferated control cells were designated as “CFSE-low” cells. Proliferation of OVA-specific DO11.10 T cells was induced by DC-mediated OVA-presentation or T cell-depleted splenocyte ( = APC) presentation of pOVA for the indicated time (B) or on day 4 (C–E). Cultures w/o antigen served as controls. Epithelial-mediated suppression was assessed in co-culture with the LA4-cell line, a murine, type II pneumocyte cell line. B: Pulmonary epithelial cells inhibit antigen-induced T cell proliferation. C: Epithelial-mediated suppression of T cell proliferation is independent from type of antigen-presenting cell. Epithelial-mediated suppression of T cell proliferation is almost entirely dependent on cell-contact of epithelial cells with T cells and DC (D) or APC (E). TC  =  T cell, DC  =  dendritic cell, OVA  =  Ovalbumin, pOVA  =  OVA-peptide, APC  =  T cell-depleted splenocyte, ns  =  not significant. *p<0.05 compared to all other variables tested or as designated by bars, calculated by Mann-Whitney-U test. 2C: ***p<0.001 1way ANOVA with Bonferroni's Multiple Comparison Test Representative experiments of n = 5–12 experiments.

Presentation of antigen within this system leads to significant T cell proliferation (measured by means of CFSE dilution – for gating strategy see Figure 2A) as early as day 4 (triangles, Figure 2B) compared to co-cultures without antigen (boxes, Figure 2B). T cell proliferation steadily increases until it reaches a plateau on day 6 where approximately 90% of all T cells proliferate. When these co-cultures were performed above a confluent monolayer of lung epithelial cells, these epithelial cells drastically reduced T cell proliferation (circles, Figure 2B) to background levels at all time points analyzed.

In order to exclude that epithelial-dependent suppression of T cell proliferation was due to the fact that the epithelial cell monolayer inhibits DC adherence to the plate bottom necessary for efficient DC antigen presentation, we additionally performed these cultures with mitomycin C-treated T cell depleted splenocytes. These APC are able to present antigen regardless of plate bottom adhesion. Direct comparisons between DC and T cell-depleted splenocytes ( = APC, Figure 2C) showed that both types of APC are able to induce a similar magnitude of T cell proliferation (1^st^ of bars, Figure 2C). Additionally, pulmonary epithelial cells inhibited T cell proliferation regardless of the type of APC used to induce T cell proliferation (2^nd^ set of bars, Figure 2C).

In a next step, we wanted to determine the contribution of cell-contact dependent mechanisms vs. soluble mediators responsible for the epithelial-dependent suppression of T cell proliferation. To this end, co-cultures of T cells and APC were separated by transwells from the pulmonary epithelial cell line. In both cases separation of the APC-driven T cell proliferation cultures from the epithelial cells almost completely restored T cell proliferation. This effect was somewhat more pronounced in T cell/DC co-cultures (3^rd^ bar, Figure 2D) than in T cell/APC co-cultures (3^rd^ bar, Figure 2E) where T cell proliferation did not completely attain levels observed in cultures without epithelial cells (1^st^ bar, Figure 2E). Thus for both types of antigen-presenting cells, cell-contact dependent mechanisms seem to be responsible for most of the epithelial-mediated suppression of T cell proliferation.

### Airway epithelial cells interfere with T cell maturation and activation but do not induce Tregs

**Figure 3:**
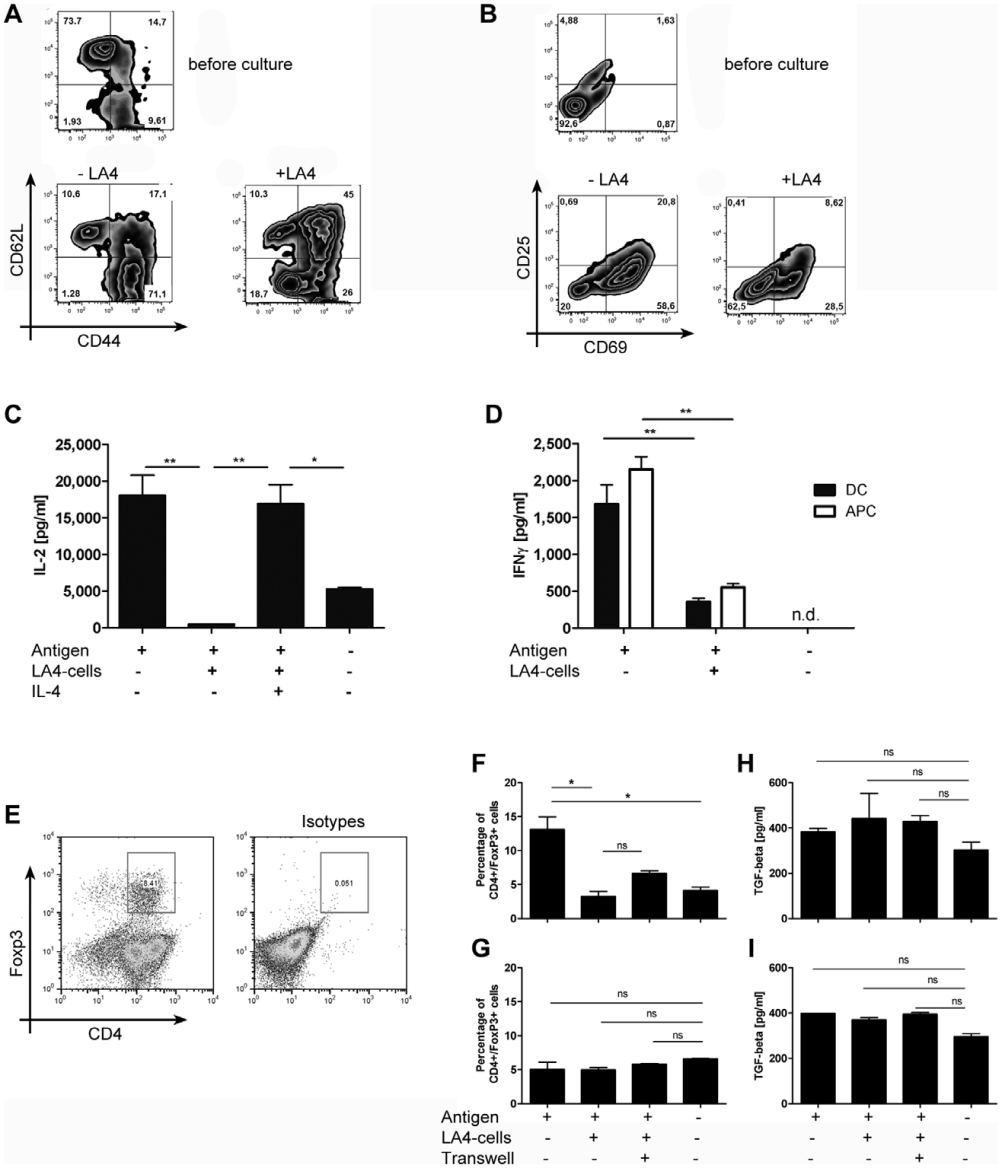
Airway epithelial cells interfere with T cell maturation and activation but not by induction of Tregs or modulation of TGF-beta secretion. Proliferation of OVA-specific DO11.10 T cells was induced by DC-mediated OVA-presentation. Cultures w/o antigen served as controls. Epithelial-mediated suppression was assessed in co-culture with the LA4-cell line, a murine, type II pneumocyte cell line. A: Expression of CD62L vs. CD44 and B: expression of CD25 vs. CD69 was assessed in co-cultures with DCs on day 4 on KJ1-26/CD4+ T cells. C: Secretion of IL-2 in supernatants on day 4 of co-culture with DC as antigen presenting cells. LA4 cells were pretreated 24 h before co-culture with 10 ng/ml IL-4. D: IFN-gamma secretion in supernatants on day 4 of co-cultures with APC or DC as antigen-presenting cells. E: Representative FACS-plot of FoxP3 staining in CD4 T cells from co-cultures of T cells with DCs. F+G: Percentage of FoxP3+/CD4+ T cells in co-cultures with DCs (F) or APC (G) on day 4. H+I: Secretion of TGF-β in co-cultures with DCs (H) or APC (I) on day 4. *p<0.05 compared to all other variables tested or as designated by bars, calculated by Mann-Whitney-U test. 3D: *p<0.05 **p<0.01 ***p<0.001 1-way ANOVA with Bonferronís Multiple Comparison Test. Representative experiments of n = 5–12.

Consecutively, we were interested to understand the effects of pulmonary epithelial cells on T cell activation in more detail. We thus analyzed T cell expression of CD62L vs. CD44 and expression of the activation markers CD25 and CD69 on day four of our co-cultures with or without pulmonary epithelial cells. At the beginning of the cultures, most of the transgenic T cell population expressed high levels of CD62L with the majority of the cells showing no expression of CD44 (Figure 3A). A small subpopulation showed expression of CD62L and CD44 and another small part of the population only expressed CD44. In summary, the majority of the T cells showed a phenotype of naïve T cells. Addition of DCs and antigen led to a shift towards increased CD44 expression with the majority of the T cells now showing CD44 expression only and smaller populations expressing CD44 and CD62L or CD62L only. Co-culture with the pulmonary epithelial cell line markedly reduced this shift: in T cell/DC co-cultures with pulmonary epithelial cells, the majority of transgenic T cells expressed CD62L and CD44 but a significant subset also expressed CD44 only and a smaller subpopulation expressed CD62L only. Interestingly, in these co-cultures, we could also observe a small but significant subpopulation of T cells which did not express CD62L or CD44. In summary, these results suggest that the pulmonary epithelial cell line arrested the course of T cell activation.

Concerning the expression of the activation markers CD69 and CD25 on day four of our co-cultures of DC and T cells, we observed an induction of both markers on day four with more than ¾ of the population expressing CD69 at this timepoint (Figure 3B) and the remaining population expressing neither marker. Pulmonary epithelial cells reduced the expression of CD69 by more than 50%, leaving a population of about 1/3 expressing CD69. This reduction of activation markers leads to a marked increase in the population which does not express any of the two activation markers, rendering almost 2/3 of the population devoid of both activation markers. In summary, these results show that pulmonary epithelial cells reduce T cell activation in its early stages, as CD69 is a classical marker of early T cell activation, being induced after less than 12 h of T cell activation [18].

In that same line, we analyzed cytokine expression by the T cells in our co-culture system. We consistently observed induction of large amounts of IL-2 in co-cultures of DCs with Ag and T cells which was nearly abrogated by LA4 cells. Pre-treatment of LA4 cells with IL-4 reduced this effect (Figure 3C). Similar to Wang et al., we observed IFN-gamma secretion in DC and APC co-cultures with Ag and T cells which was also reduced by LA4 cells (Figure 3D). We were not able to detect significant amounts of IL-4 or −5 in the co-culture supernatants (data not shown) suggesting that the T cell phenotype induced was leaning towards a Th1 phenotype. One possible explanation for the suppressive effects of pulmonary epithelial cells is the induction of Tregs as it has been suggested by other studies [13], [19]. However, we were not able to reproduce these findings. Measuring the percentage of CD4+FoxP3+ T cells in our co-culture system by means of flow cytometry (for gating strategy see Figure 3E), in our hands T cell activation by antigen-presenting DC led to a significant increase in FoxP3+ T cells from 4.14% in co-cultures of DC and T cells without antigen (4^th^ bar, Figure 3F) to 13.1% in co-cultures with antigen added (1^st^ bar, Figure 3F). Co-culture on an epithelial cell monolayer led to a reduction, rather than an increase of FoxP3+ cells to 3.25% (2^nd^ bar, Figure 3F) which was not significantly changed by separation of T cell/DC cultures from epithelial cells by means of transwell inserts (3^rd^ bar, Figure 3F). In co-cultures of T cells with T cell-depleted splenocytes we could not observe the increase in FoxP3+ T cells by antigen-presentation (1^st^ bar, Figure 3G) compared to cultures without antigen presentation (4^th^ bar, Figure 3G) which we observed in the T cell/DC co-culture system. Yet, similarly to the results observed in the T cell/DC system, co-culture of T cell/APC on pulmonary epithelial cells did not increase the percentage of T cells expressing FoxP3 (2^nd^ bar, Figure 3G) suggesting that independently of the type of antigen-presenting cell, increases in regulatory T cells are not responsible for the suppression of T cell activation which we observed in our co-culture systems. In line with these findings, we could not detect any change in TGF-beta concentration, an immunomodulatory cytokine, that is described to induce Foxp3 expression in T cells [20], in supernatants from T cell/DC (Figure 3H) or T cell/APC (Figure 3I) co-cultures, regardless of the conditions tested.

### Suppression of T cell proliferation by airway epithelial cells is attenuated by IL-4

**Figure 4:**
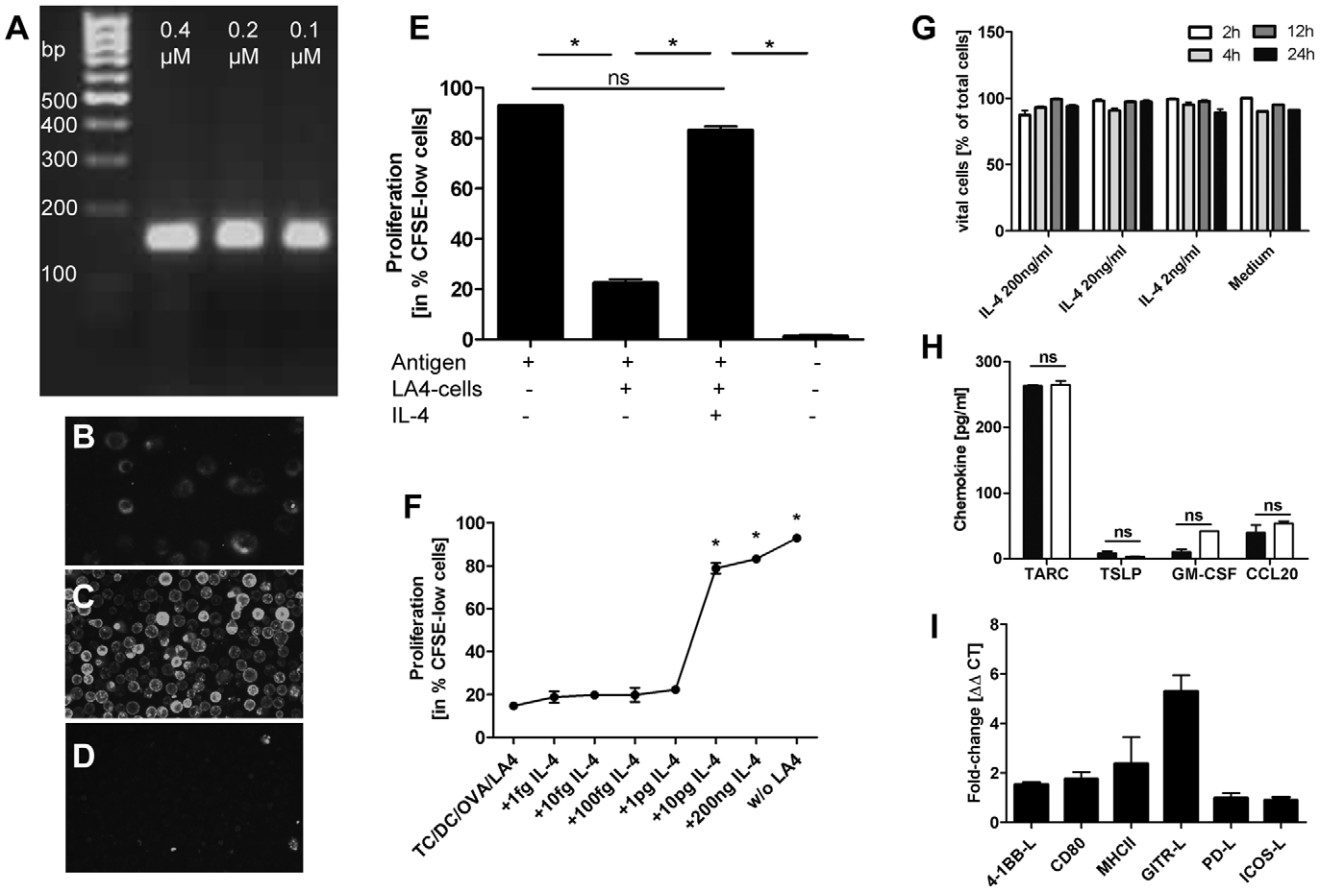
Suppression of T cell proliferation by airway epithelial cells is attenuated by IL-4. Proliferation of OVA-specific DO11.10 T cells was induced by DC-mediated OVA-presentation. Cultures w/o antigen served as controls. Epithelial-mediated suppression was assessed in co-culture with the LA4-cell line, a murine, type II pneumocyte cell line which was pre-treated with the indicated doses of IL-4 (F,G) or pre-treated with 200ng (E) or 10pg of IL-4 (H+I). Harvests were performed on day 4 of co-culture (E+F) or 24 h after IL-4 treatment (H+I). A: mRNA expression of IL-4Rα on untreated LA4 cells, shown are products obtained with different primer concentrations. B: Assessment of IL-4Rα surface expression via immunofluorescence microscopy of untreated LA4 cells, C: Corresponding IL-4Rα surface expression on splenocytes cultured with 1 µg ConA for 3d, D: Corresponding isotype control antibody. E: Pre-treatment with IL-4 (200 ng) reduces epithelial mediated suppression of T cell proliferation on day 4 of co-culture. F: Attenuation of epithelial-dependent suppression of T cell proliferation by IL-4 is dose-dependent. A culture without LA-4 cells (w/o) and IL-4 served as control. G: IL-4 treatment for 2–24 h at the indicated doses does not influence viability of LA4 cells, measured by trypan blue exclusion. H: IL-4-treatment of epithelial cells (10 pg; white) does not change epithelial cytokine secretion (black). I: IL-4 induces GITR-L expression in epithelial cells. TC  =  T cell, DC  =  dendritic cell. *p<0.05 compared to all other variables tested or as designated by bars, calculated by Mann-Whitney-U test. Representative experiments of n = 3–4.

After having explored the key aspects of epithelial-mediated suppression of T cell activation, we used this system to analyze the effect of a pre-treatment of the epithelial cell line with IL-4. In initial experiments we established that the pulmonary epithelial cell line used does express the IL-4Rα as a prerequisite for IL-4 dependent airway priming in our in vivo system (compare Figure 1). RT-PCR with primers specific for the IL-4Rα (CD124) demonstrated for the first time that the pulmonary epithelial cell line we used does indeed express the IL-4Rα (Figure 4A) ensuring that a response towards IL-4 by this cell line is indeed possible. Similarly, we were able to detect expression of the IL-4Rα protein on the cell surface of the LA4 cell line used. Immunohistochemistry with appropriate antibodies and isotype controls showed a low, but well discernible expression of CD124 on LA4 cells (Figure 4B) compared to appropriate isotype control staining (Figure 4D). Splenocytes stimulated with 1µg of ConcanavalinA served as positive controls which showed a more pronounced expression (Figure 4C) compared to the pulmonary epithelial cell line LA4.

We consecutively treated a confluent layer of the epithelial cell line with 200 ng of IL-4 for 24 h which we had extrapolated from doses of IL-4 used in the literature. The epithelial cell line monolayer was subsequently washed thoroughly before T cells, DC and antigen were added. We observed that pre-treatment of the epithelial cell line with 200 ng of IL-4 almost completely restored T cell proliferation to the levels observed in T cell/DC co-cultured without the pulmonary epithelial cell line (3^rd^ bar vs. 1^st^ bar, Figure 4E). Consecutively, we analyzed dose-response curves which showed that a dose of 10pg/ml was sufficient to inhibit epithelial-mediated suppression of T cell proliferation (Figure 4F).

Since Wang et al. described increased epithelial cell death after RSV infection as a possible contributor to the effects they observed in their co-culture system [13], we addressed the effect of different doses of IL-4 on epithelial viability by analysis of trypan blue exclusion after 2–24 h. As can be observed in Fig. 4G, neither concentration of IL-4 led to changes in LA4 viability compared to LA4 cells cultured in medium alone, excluding effects of IL-4 on epithelial cell viability as a major contributor to the effects we observed.

Additionally, we analyzed supernatants of LA4 cells stimulated for 24 h with 10pg/ml of IL-4 for changes in TARC, TSLP, GM-CSF and CCL20 secretion as modulation of these soluble mediators has been shown to underlie immunomodulatory mechanisms exerted by epithelial cells. However, we were not able to observe changes in secretion of any of these markers induced by treatment with IL-4 (Figure 4H). These results are in concurrence with our transwell experiments though, where we observed that cell-contact mediated mechanisms play a crucial role, making changes in soluble mediators as an important mechanism for the alterations induced by IL-4 a rather unlikely explanation. In order to identify cell-contact mediated changes induced by treatment with IL-4, we analyzed variations in costimulatory molecule expression on the epithelial cell line which have been associated with immunomodulation by epithelial cells in independent studies. When analyzing the relative expression levels of MHCII, CD80, PD-L, 4-BBL and GITR-L by RT-PCR after 24 h of culture with 10pg/ml IL-4, we could observe an almost 6-fold upregulation of mRNA expression of GITR-L but not of the other molecules tested (Figure 4I). These results suggest that increases in GITR-L surface expression might contribute to IL-4 mediated modulation of epithelial cell function, a hypothesis which remains to be formally tested.

## Discussion

To our knowledge the data we present here are the first to demonstrate that epithelial-mediated suppression of T cell activation is inhibited by IL-4, a key cytokine in allergic airway disease. We had previously shown that IL-4 dependent induction of a Th2-polarized airway inflammation equally depends on the hematopoetic and the structural compartment's ability to respond to IL-4 [16]. This knowledge is extended by our data, as they demonstrate that epithelial cells which constitute an important part of the structural compartment within the airways, are modulated in their suppressive phenotype by IL-4. Furthermore, our data enhance our knowledge on epithelial-mediated suppression of T cell proliferation as they show that co-culture with epithelial cells prevents early T cell activation keeping them in a naïve-like state. Together, these data could serve as an explanation for the increased risk for consecutive sensitizations to new allergens we observe in (mono-) sensitized individuals: the Th2-polarized airway inflammation these individuals experience, particularly upon contact with antigen leads to the secretion of high levels of IL-4 within the airways [21] which, if acting in a similar fashion, as we observed in our in vitro system, would allow increased T cell activation in response to other antigens encountered in the airways and thus lead to facilitated priming towards new antigens and ultimately to polysensitization.

Cell lines do not necessarily reflect effects exerted by their primary *in vivo* counterparts. We used a cell line of epithelial cells originating from mouse lung adenoma for our in vitro experiments. This cell line was extensively analyzed regarding its morphology by Stoner and colleagues and described to closely resemble type II alveolar epithelial cells [17]. Functionally, we could show the secretion of CCL20, GM-CSF, TARC and TSLP (Figure 4H) as well as expression of MHCII and different co-stimulatory molecules (Figure 4I) by LA4 cells; attributes which have been described for primary type II cells [22], [23]. Wang et al. performed a detailed comparison between LA4 cells vs. primary type II pneumocytes with regards to their effects on T cell activation [13] and could show very similar responses for both cell types. We thus believe, that an extrapolation from our data obtained with the LA4 cell line, which retrace the results from Wang et al., to primary type II pneumocytes is cautiously justified.

Our experiments did not directly address whether the effects exerted by the epithelial cell line were due to direct effects on DCs or on T cells. With regards to direct effects of the LA4 cell line on DCs, Wang et al. could already show that T cell inhibition by LA4 cells was also observed if the T cell activation was induced by CD3/CD28 antibodies, rendering a direct effect of the epithelial cells on DCs rather unlikely. Our own results, which show that epithelial inhibition of T cell proliferation takes place with different antigen presenting cells (DC vs. APC), underlines that point. Since we performed extensive washing of the IL-4 treated LA4 cells before co-culture with DCs (and T cells), we believe that we can exclude a direct effect of the IL-4 on DCs. In order to exclude direct effects of miniscule remaining doses of IL-4 on T cells after the washes, we included appropriate controls with T cells co-cultured with DCs and IL-4 alone which consistently failed to result in T cell activation or proliferation (data not shown).

Our experiments could not recapitulate all of the published data pertaining to the mechanisms underlying epithelial-mediated inhibition of T cell proliferation, though. For instance Wang et al. but also Gereke et al. could show that pulmonary epithelial cells promote the differentiation of FoxP3+ regulatory T cells [13], [19]. We were not able to confirm these data in our system, neither with DC nor with T cell-depleted splenocytes as antigen-presenting cells. Some of these differences might be due to the type of epithelial-cell used and the experimental approach: Gereke et al. using conditioned medium from primary epithelial cells isolated from mouse lungs suggesting that soluble mediators play a major role in their system. However, Wang et al. used the same epithelial cell line as we employed and still demonstrated different results regarding the induction of regulatory T cells. Yet, their purification for T cells but particularly the protocol for generation of BMDC was different from ours in that they used BMDC after a longer culture period. In that line, others have shown that uptake of apoptotic or necrotic DCs by other DCs leads to a tolerogenic phenotype with Treg-inducing quality [24]. Modulation of the DC-capacity to induce Tregs by this mechanism could underlie the differences, we observed compared to Wang et al. Finally, many studies have shown changes in phenotypes in cultured cells lines. We cannot exclude that due to repeated passage our epithelial cell line has developed phenotypical changes which alter its properties compared to the cell line Wang et al. used.

In that same line, we not only observed differences with regards to the Treg-inducing capacities of the LA4 cells, but also with regards to cell-contact dependency of the immunomodulatory effects, exerted by the LA4 cell line. We found almost complete dependency on cell-contact and were not able to show differences in TGF-beta secretion in co-cultures of LA4 cells with T cells and DCs or T cells and APC vs. co-cultures without LA4 cells. In contrast Wang et al. showed pronounced but not exclusive cell-contact dependency and a contribution of TGF-beta as an important soluble mediator. In this respect, our data are more in agreement with Cruickshank et al. who demonstrate that colonic epithelial cells retain T cells in the G1 phase of cell cycle by a cell-contact dependent and TGF-β independent mechanism [12]. In our hands LA4 cells kept T cells in a naïve-like state in both DC-and APC-driven cultures which could be explained by a cell cycle arrest such as observed by Cruikshank et al.

In their study Wang et al. not only studied mechanisms underlying epithelial-mediated T cell suppression overall but went on to characterize the effects of an infection of the epithelial cells with RSV, a respiratory virus often associated with an increased prevalence of allergic sensitization and the development of asthmatic symptoms. They showed that RSV-infection of epithelial cells reduces epithelial-mediated T cell suppression similar to the effects, we observed for treatment of epithelial cells with IL-4. Interestingly, RSV infection has been associated with an increase in Th2 cytokine secretion [25]. In their study Wang et al. did not assess the production of Th2 cytokines by the T cells within the co-culture system. It is tempting to speculate that the effects observed for RSV might be, in parts, due to a feedback loop. RSV-infection of epithelial cells has been shown to promote secretion of Th2-skewing chemokines from epithelial cells [26]–[28]. Consecutively, increased Th2 polarization would lead to increased IL-4 secretion by T cells in the co-culture system which in turn could decrease epithelial-mediated suppression of T cell proliferation. This hypothesis might reconcile our findings with regards to the mechanisms underlying epithelial-mediated suppression of T cell suppression with Wang's. It is possible that the direct effect of RSV is mediated via TGF-beta and increased IL-4 secretion making it cell-contact independent. The effect of IL-4 itself, though could be mediated via a cell-contact dependent mediator, such as the increased expression of GITR-L, as we observed, making this part of the RSV-effect cell-contact dependent.

GITR-L is a cell surface receptor and belongs to the tumor necrosis factor receptor superfamily, which exert a multitude of different effects in many different immunological pathologies. Different studies have shown ligation of GITR to be involved in T cell activation and survival and the prevention of T cell death [29]. While expression of GITR-L had initially been described on dendritic cells [30], more recent studies revealed expression on epithelial cells in different body compartments. Interestingly, in the eye, expression of GITR-L on retinal pigment epithelial cells reduces their immunosuppression of CD3+ T cells normally found at this location, most likely by reducing TGF-beta secretion and leads to the secretion of different pro-inflammatory cytokines and chemokines by these T cells [31]. In that same line ligation of GITR-L on keratinocytes in a keratinocyte-T cell co-culture system led to increased T cell attracting chemokine secretion by the keratinocytes and GITR-GITRL ligation in this system augmented keratinocyte-induced T cell proliferation [32]. These studies support our hypothesis, that IL-4 dependent induction of GITR-L might be a mechanism by which IL-4 reduces epithelial-mediated T cell suppression, a hypothesis which needs to be tested by further experiments.

Taken together, our data show for the first time that IL-4 reduces epithelial-mediated suppression of T cell proliferation. This mechanism serves to explain the increased risk of patients with a pre-existing allergy towards developing consecutive allergies against novel allergens as their pre-existing sensitization leads to increases in IL-4 secretion by primed T cells upon each allergen-contact [21]. Furthermore, our data suggest that controlling the Th2-polarized airway inflammation in bronchial asthma in a mono-sensitized individual remains a crucial goal not only in terms of symptom-control but also towards the prevention of further allergies.
